# Dual Topology of the Melanocortin-2 Receptor Accessory Protein Is Stable

**DOI:** 10.3389/fendo.2016.00096

**Published:** 2016-07-18

**Authors:** Zachary J. Maben, Sundeep Malik, Liyi H. Jiang, Patricia M. Hinkle

**Affiliations:** ^1^Department of Pharmacology and Physiology, University of Rochester Medical Center, Rochester, NY, USA

**Keywords:** accessory protein, ACTH, biotin ligase, cAMP, MC2 receptor, MRAP, membrane protein topology, ubiquitin

## Abstract

Melanocortin 2 receptor accessory protein (MRAP) facilitates trafficking of melanocortin 2 (MC2) receptors and is essential for ACTH binding and signaling. MRAP is a single transmembrane domain protein that forms antiparallel homodimers. These studies ask when MRAP first acquires this dual topology, whether MRAP architecture is static or stable, and whether the accessory protein undergoes rapid turnover. To answer these questions, we developed an approach that capitalizes on the specificity of bacterial biotin ligase, which adds biotin to lysine in a short acceptor peptide sequence; the distinct mobility of MRAP protomers of opposite orientations based on their *N*-linked glycosylation; and the ease of identifying biotin-labeled proteins. We inserted biotin ligase acceptor peptides at the *N*- or C-terminal ends of MRAP and expressed the modified proteins in mammalian cells together with either cytoplasmic or endoplasmic reticulum-targeted biotin ligase. MRAP assumed dual topology early in biosynthesis in both CHO and OS3 adrenal cells. Once established, MRAP orientation was stable. Despite its conformational stability, MRAP displayed a half-life of under 2 h in CHO cells. The amount of MRAP was increased by the proteasome inhibitor MG132 and MRAP underwent ubiquitylation on lysine and other amino acids. Nonetheless, when protein synthesis was blocked with cycloheximide, MRAP was rapidly degraded even when MG132 was included and all lysines were replaced by arginines, implicating non-proteasomal degradation pathways. The results show that although MRAP does not change orientations during trafficking, its synthesis and degradation are dynamically regulated.

## Introduction

The hypothalamic/pituitary/adrenal axis comprises a classical endocrine loop in which hypothalamic corticotropin-releasing hormone stimulates synthesis and release of ACTH from corticotropes of the anterior pituitary gland, ACTH stimulates glucocorticoid production in the adrenal gland, and glucocorticoids exert feedback control centrally. ACTH stimulates multiple steps in the glucocorticoid biosynthetic pathway, acting via a receptor coupled to the heterotrimeric Gs protein to activate adenylyl cyclase and elevate intracellular cAMP. ACTH also exerts receptor-dependent but cAMP-independent effects (1).

The receptor for ACTH was identified almost 25 years ago by Mountjoy et al. (2). The melanocortin 2 (MC2) (ACTH) receptor is one of five structurally related G protein-coupled melanocortin receptors, MC1 through MC5, all of which are activated by peptides derived from pro-opiomelanocortin, or POMC. The MC2 receptor was found to differ from the other four melanocortin receptors in two important ways. First, it could not be activated by α-, β-, or γ-MSH but only by the longer ACTH peptide. Second, it was not functional unless it was expressed in adrenal or melanoma cells. This led to speculation that the MC2 receptor required an additional protein present in a limited number of cell types.

The nature of the hypothetical accessory protein remained a mystery for more than a decade, when Metherell et al. (3) identified the gene encoding MC2 receptor accessory protein (MRAP) from individuals with familial glucocorticoid deficiency type 2, which is characterized by resistance to ACTH despite normal MC2 receptor genes. MRAP encodes a small protein with a single predicted transmembrane domain. Two splice variants of human MRAP (α and β) are identical in the aminoterminal and transmembrane regions. When MRAP was co-expressed with MC2 receptor, the receptor was able to traffic to the plasma membrane and respond to ACTH with an increase in cAMP in cells that were otherwise non-responsive. MRAP and MC2 receptors have been shown to interact closely by a variety of approaches including co-precipitation (4, 5), fluorescence microscopy (6, 7), bimolecular fluorescence complementation (8), and co-internalization in response to ACTH (9). Recent data establish that two MRAP dimers can interact with one MC2 receptor and that two MC2 receptors can interact with one MRAP, but it is unclear whether this happens normally (10). MRAP2, an MRAP paralog, co-precipitates with the MC2 receptor and all other members of the melanocortin receptor family (5) but does not support signaling by MC2 receptors (11).

Using an alanine-scanning mutagenesis approach and focusing on the highly conserved aminoterminus of MRAP, we discovered that MRAP lacking a critical tyrosine-rich region was able to promote MC2 receptor trafficking but not signaling (11). This established that MRAP has two distinct functions, one to enable MC2 receptor trafficking to the cell surface, perhaps because it assists protein folding, and a second to enable signal transduction. Importantly, when MC2 receptors were expressed with a mutated MRAP, receptors were localized on the plasma membrane but completely unable to bind ACTH. As a result, ACTH cannot be expected to exert any effect – cAMP-dependent or otherwise – in cells lacking MRAP.

It is well established that mRNA encoding MC2 receptor undergoes feed-forward regulation, increasing in response to stress or ACTH (12). MRAP mRNA levels also increase, and do so within minutes, in response to either an imposed stress or ACTH (13, 14). MC2 receptor and MRAP mRNAs rise dramatically when cultured adrenal cells are incubated with ACTH, establishing a direct action at the adrenal gland (15). It is not known whether MC2 receptor and MRAP protein levels also change on a rapid time scale. One of the goals of the experiments described here was to characterize MRAP turnover in an isolated cell system.

The architecture of MRAP is extremely unusual. MRAP appears to form antiparallel homodimers. This conclusion is based on the following: (1) immunological findings showing dual topology, where antibodies identify both the amino and carboxyterminal ends of MRAP facing outwards on the cell surface (4); (2) quantitative co-precipitation of differentially tagged MRAPs, pointing to a multimeric structure (4); (3) biochemical experiments showing that approximately half of MRAP undergoes glycosylation at the single predicted site for *N*-linked glycosylation, a process that takes place in the interior of the endoplasmic reticulum (ER) and Golgi apparatus (4); (4) bimolecular fluorescence complementation (8) and bioluminescence resonance energy transfer (16) in configurations consistent with an antiparallel dimeric structure; and (5) evidence that a concatenated protein made by fusing two MRAPs to the extracellular aminoterminus of the MC2 receptor is ACTH-responsive (10). The latter studies established that the tyrosine-rich domain of the MRAP aminoterminus is necessary on the outer surface of the cell, likely allowing ACTH to bind.

Taken together, these data provide strong evidence that MRAP forms antiparallel homodimers. MRAP and MRAP2 are the only single transmembrane domain proteins thought to exist in this configuration. It is not yet known when MRAP conformation is established, whether MRAP orientation changes during synthesis and trafficking, whether dual topology predominates in adrenal cells, and whether MRAP is stable. We designed the experiments reported here to address these unresolved issues and describe several approaches to probe the topology of the MRAP protein at different times after biosynthesis and estimate its half-life.

## Materials and Methods

### Reagents

Melanocortin 2 receptor accessory protein plasmids that have not been described previously (4, 8, 11) were constructed using QuikChange from Stratagene with standard molecular biological techniques and verified by sequencing. MRAP–V5–3xFlag contained mMRAP followed by GKPIPNPLLGLDSTGRDYKDHDGDYKDHDIDYKDDDDK at the C-terminus. AP–MRAP contained the minimal AP sequence GLNDIFEAQKIEWHE immediately after the initiating Met followed by mMRAP or mMRAP–V5–3xFlag. MRAP–AP contained the same AP sequence at the C-terminus of mMRAP or mMRAP–V5–3xFlag followed by a stop codon. MRAPs containing the AP sequence but no epitope tags were constructed similarly. On figures, these constructs are shown as AP–MRAP or MRAP–AP and epitope tags are described in the legends. V5–MART1–AP and AP–MART1–V5 were constructed by adding V5 epitope (GKPIPNPLLGLDST) and AP sequence (GLNDIFEAQKIEWHE) to opposite ends of mMART1. (4K to R)MRAP refers to V5–mMRAP–3xFlag with the four Lys in native mMRAP mutated to Arg. 3xHA-hMC2 receptor, referred to as MC2 receptor, was from Missouri cDNA Resource Center and plasmid encoding eGFP from Clontech. BirA in pcDNA3 and BirA_ER_ in pDisplay vectors were generously donated by Dr. Alice Ting (Massachusetts Institute of Technology) and CRE-luciferase by Dr. George Holz (SUNY Upstate Medical University). Plasmid encoding HA-ubiquitin (HA-Ub) has been described (17). Monoclonal antibodies were from the following: Sigma (M2 anti-Flag and immobilized M2 anti-Flag), Covance (HA-11 anti-HA), and AbDSerotec (anti-V5). Streptavidin–HRP and streptavidin–agarose were from Thermo Scientific, immobilized Protein A/G beads from Santa Cruz Biotechnology, and HRP-labeled antibody against mouse heavy and light chains from BioRad.

### Cell Growth and Transfection

CHO cells were from American Type Culture Collection and OS3 adrenal cells (18) from Dr. Bernard Schimmer (University of Toronto). CHO and OS3 cells were maintained in DMEM/F12 medium containing 5% fetal bovine serum at 37°C in 5% CO_2_-95% air and passaged with trypsin. Transfection was performed using FugeneHD or Lipofectamine 3000 according to manufacturers’ instructions. Stable cell lines were generated by transfecting with plasmids encoding an MRAP, BirA, or BirA_ER_ and selecting with 1 mg/ml G418. Pooled cells were used for experiments. Biotin-labeling experiments were performed by adding 2.5 μM biotin to complete medium at the time of transfection unless otherwise noted.

### cAMP Responses

In brief, cells were grown in white 96-well plates and transfected with 40–50 ng total DNA/well, usually with equal parts of MC2 receptor, MRAP construct and CRE-luciferase, a reporter containing multiple cAMP response elements from the rat insulin promoter (19). After 24 h cells were challenged for 4–5 h with hACTH(1–24) or 20 μM forskolin in DMEM with 0.1% BSA. Medium was replaced with Firefly Reagent from Nanolight and luminescence read in a BioTek platereader. Unless noted, results are expressed as percent of the forskolin response measured in the same experiment.

### Immunoprecipitation, SDS-PAGE, and Immunoblotting

Cells were washed with PBS and lysed on ice in Tris/Mg/EGTA buffer (150 mM NaCl, 50 mM Tris-Cl, 1 mM EDTA, and 1% Triton X-100, pH 8.0) containing protease inhibitors. Iodoacetamide (10 mM) was included in lysis buffers when ubiquitin labeling was being assessed. Samples were centrifuged at 10,000 × *g* for 20 min to remove nuclei and supernatants used for analysis. In some cases, HA- or Flag-labeled proteins were immunoprecipitated by incubating overnight at 4°C with 1:1000 dilutions of antibody and collected on Protein A/G beads. Biotin-labeled proteins were pelleted following overnight incubation with streptavidin–agarose beads. Samples were taken up in NuPage LDS sample buffer with a final concentration of 50 mM dithiothreitol. For analysis by SDS-PAGE samples were run on Lonza PagR or BioRad TGX gels. Proteins were transferred to nitrocellulose, blocked in TBST (Tris-buffered saline with 0.05% Tween 20) with 5% non-fat dry milk, and incubated overnight at 4°C in either monoclonal anti-V5, anti-HA, or anti-Flag antibody at 1:5000 in TBST/milk. After washing, antibody blots were incubated in 1:5000 HRP-anti-mouse heavy and light chain, washed and visualized using Western Lightning chemiluminescent reagent. To visualize biotin-labeled proteins, blots were incubated in 1:10,000 HRP–streptavidin in TBST with 0.5–1% BSA, because milk contains biotin, washed and developed. In some cases, blots were subsequently incubated in 0.1% sodium azide to inactivate HRP and blotted with antibody as described above.

### Cell Surface ELISA

Expression of proteins on the plasma membrane was quantified as described previously (4, 11). Cells grown in 12- or 24-well plates were fixed with 2 or 3% paraformaldehyde for 10–20 min at room temperature, washed with PBS, and incubated for ~1 h with 1:5000 monoclonal anti-HA, anti-V5, or anti-Flag antibodies in PBS containing 5% non-fat dry milk. Cells were then washed extensively and incubated for ~1 h with 1:5000 HRP-labeled anti-mouse IgG, and washed prior to the addition of tetramethylbenzidine substrate (Sigma) and measurement of absorbance at 450 nm.

### Fluorescence Microscopy

For microscopy, CHO cells stably expressing BirA_cyt_ or BirA_ER_ were grown on glass coverslips and transfected with AP-tagged MRAP constructs. After overnight incubation, live cells were incubated with anti-Flag antibody (1:250 in serum-containing medium) for 30 min at 37°C, washed, and then incubated with 1:500 Alexa488-labeled anti-mouse M2 and 1:500 Alexa555-labeled streptavidin and incubated for 30 min at room temperature. Cells were washed, fixed in paraformaldehyde, washed again and mounted in Prolong Gold (InVitrogen), and viewed in a Nikon epifluorescence microscope with a 40× oil objective. Images showing Flag and streptavidin were obtained using 500 and 2500 ms exposures, respectively. All Flag and all streptavidin micrographs were processed identically.

### Miscellaneous

To deglycosylate proteins, cell lysates were denatured and incubated with PNGaseF from New England Biolabs following manufacturer’s instructions; control lysates were incubated identically in reactions lacking enzyme. All experiments were repeated a minimum of two times. Error bars indicate mean and range or SE of duplicate or triplicate determinations in representative experiments. Significance of differences between two values was determined by Student’s *t*-test.

## Results

### When Is MRAP Topology Established?

**Figure 1:**
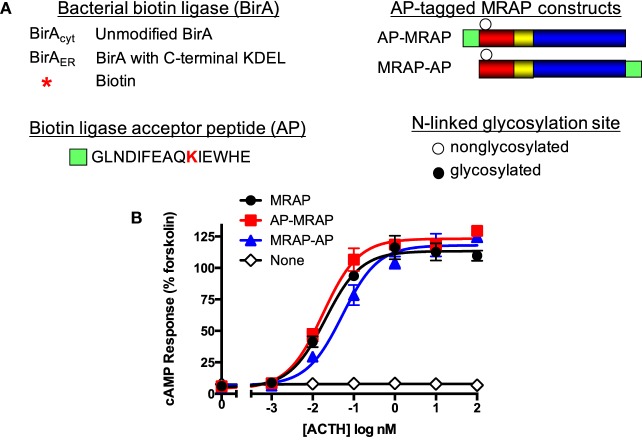
**Use of biotin tagging to discern MRAP orientation**. **(A)** Schematic representation showing the sequence of the acceptor peptide (AP) for bacterial biotin ligase that catalyzes addition of biotin to Lys. Addition of C-terminal KDEL sequence to BirA targets it to the ER. Circles depict a potential *N*-linked glycosylation site (Asn-X-Ser/Thr) with or without glycosylation. An AP sequence was added immediately after the initiating Met in AP–MRAP or at the C-terminus in MRAP–AP as detailed in Section “Materials and Methods.” **(B)** ACTH responses of CHO cells expressing a cAMP-responsive CRE-luciferase reporter, MC2 receptor, and MRAP constructs.

The strategy used to determine whether MRAP is first synthesized in dual orientations is outlined schematically in Figure 1A. Biotin labeling of a protein modified to contain a decarboxylase biotin acceptor domain was used previously to characterize membrane insertion of bacterial lactose permease (20). The biotin-labeling approach described here uses a small acceptor sequence and exogenous BirAs to provide a new means to analyze MRAP architecture. The bacterial enzyme biotin ligase (BirA) attaches a biotin residue to the single Lys residue in a specific acceptor peptide sequence (AP). When expressed in mammalian cells, bacterial biotin ligase is localized in the cytoplasm; addition of a carboxyterminal KDEL sequence targets the enzyme to the ER (21, 22). Because there are relatively low concentrations of biotin in standard serum-supplemented tissue culture media, it is straightforward to initiate biotin labeling of a protein tagged with an AP sequence by adding exogenous biotin. Mouse MRAP contains a single *N*-linked glycosylation site at Asn3 of the aminoterminus and *N*-glycosylation occurs exclusively in the inside of the ER and Golgi apparatus, which is topologically equivalent to the exoplasmic face of the plasma membrane. Glycosylated MRAP, which migrates more slowly than non-glycosylated MRAP on SDS-PAGE, must, therefore, be in the N_exo_/C_cyt_ orientation unless it has changed orientations during trafficking. We engineered MRAPs with an AP sequence at either the aminoterminal or carboxyterminal end of the protein; each of these MRAP constructs also contained a Flag epitope. As shown in Figure 1B, control MRAP, AP–MRAP (*N*-terminal AP sequence), and MRAP–AP (C-terminal AP sequence) all supported comparable ACTH-stimulated increases in a cAMP reporter.

**Figure 2:**
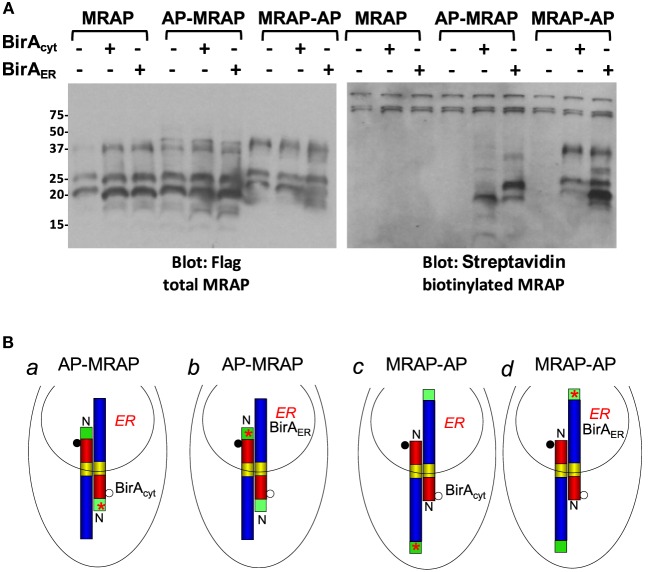
**Biotin labeling confirms dual topology of MRAP**. **(A)** CHO cells stably expressing BirA_cyt_ or BirA_ER_ were transfected with MRAP, AP–MRAP, or MRAP–AP; these plasmids also contained a Flag epitope. Following detergent solubilization, proteins were resolved on SDS-PAGE and probed with monoclonal anti-Flag antibody and HRP-anti-mouse IgG or HRP–streptavidin. **(B)** Expected localization of BirA and predicted orientation of newly synthesized MRAPs. Filled and open circles depict glycosylated and non-glycosylated MRAP and red stars represent biotin.

**Figure 3:**
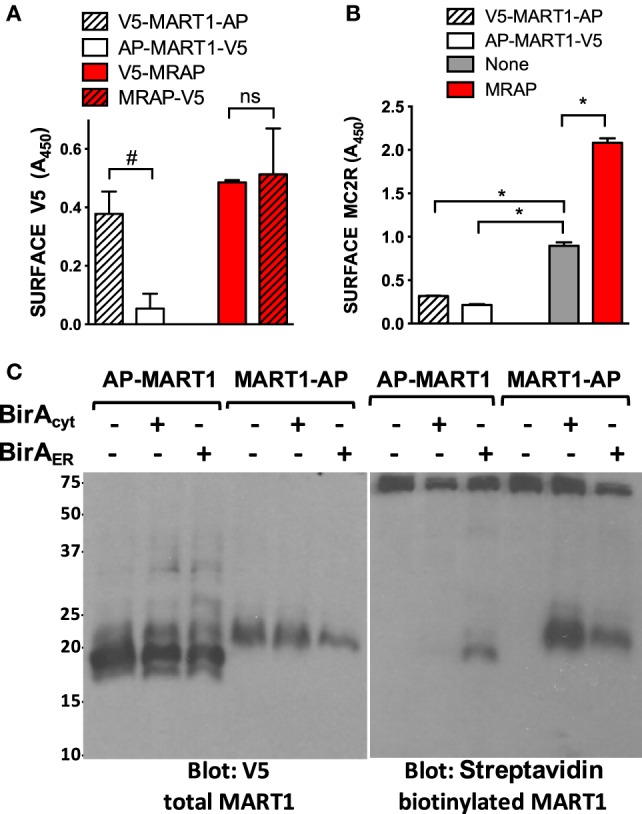
**Biotin labeling confirms single orientation of a control protein**. **(A)** CHO cells were transfected with plasmids encoding MRAP or MART1 with AP and V5 epitopes at the N- or C-terminus as shown. The topology prediction programs TMHMM and HMMTOP predict that both unmodified and epitope-tagged versions of MART1 will assume an exclusively N_exo_ orientation whereas MRAPs are likely to assume both N_exo_ and N_cyt_ orientations. **(B)** Cells were transfected with plasmids encoding HA-tagged MC2 receptor and MRAP or MART1 as in **(A)** and surface receptor was quantified. Expression of tagged proteins on the plasma membrane was measured as described in Section “Materials and Methods.” **(C)** Plasmids encoding MART1 tagged with AP at either the N- or C-terminus and V5 epitope at the opposite end were transfected into CHO cells stably expressing BirA_cyt_ or BirA_ER_. High MW bands seen in all lanes are endogenous biotinylated proteins. *P < 0.05; #*P* < 0.1.

MRAP, AP–MRAP, and MRAP–AP were expressed in CHO cells stably expressing either cytoplasmic BirA or ER-targeted BirA_ER_. The upper left panel of Figure 2 shows a Flag immunoblot identifying total MRAP. In every case, monomeric glycosylated and non-glycosylated MRAP species ran as a doublet at ~20 and 25 kDa. Enzymatic degradation with the glycosidase PNGaseF confirmed that the two major bands represented MRAP with and without *N*-linked glycosylation (4) and Figure 11 below. Higher MW bands are likely MRAP multimers and their mobility shifts following PNGaseF treatment (not shown). The upper right panel of Figure 2A shows the same blot probed with streptavidin to detect only biotin-labeled proteins. Despite having identical molecular weights, AP–MRAP and MRAP–AP consistently displayed different mobilities on SDS-PAGE, as do AP–MART1 and MART1–AP (Figure 3 below). It is possible that the proteins undergo different post-translational modifications but the behavior of membrane proteins on SDS-PAGE is not always predictable.

Endogenous biotinylated carboxylases are visible in all lanes at high molecular weight (75 kDa and above) in the blot on the upper right. These metabolically labeled proteins do not depend on transfected BirA. The three control lanes shown on the left of each blot in Figure 2 demonstrate that MRAP lacking the biotin acceptor peptide was expressed well but not labeled with biotin. Cytoplasmic BirA catalyzed the addition of biotin to the faster-migrating, non-glycosylated form of AP–MRAP (situation *a* in Figure 2B), whereas ER-targeted BirA_ER_ added biotin to the higher molecular weight glycosylated form of AP–MRAP (situation *b*). The reverse was found when cytoplasmic BirA and ER-targeted BirA_ER_ were expressed with MRAP–AP. In this case, BirA_cyt_ preferentially labeled glycosylated MRAP–AP and BirA_ER_ preferentially labeled non-glycosylated MRAP–AP (situations *c* and *d*, respectively). These results support the conclusion that the great majority of MRAP molecules achieve an antiparallel orientation when first synthesized. The finding of some biotin-labeled but non-glycosylated AP–MRAP with BirA_ER_ could result from (1) biotin labeling of AP–MRAP that is incompletely glycosylated, (2) incomplete import of overexpressed BirA_ER_ into the ER leaving some active enzyme in the cytoplasm, or (3) a change in orientation of a small portion of MRAP after synthesis. The finding of a biotin-labeled and glycosylated MRAP–AP band with BirA_ER_ is consistent with incomplete BirA_ER_ import. After overnight incubation in biotin-supplemented media, essentially all AP-tagged MRAP could be collected on streptavidin–agarose beads, implying nearly quantitative biotinylation.

As an additional control for this approach, we studied MART1 (also called Melan-A), a melanosome protein with a single transmembrane domain, strongly predicted N_exo_/C_cyt_ orientation, and size close to that of mouse MRAP (18 vs. 14 kDa without epitopes or carbohydrate). We engineered plasmids encoding MART1 with AP tags at one end or the other and V5 tags at the opposite ends. We used a previously described fixed cell ELISA protocol (4) in which antibodies were added to intact cells to detect epitopes localized on the outside of the plasma membrane (Figure 3A). MART1 was found in the predicted N_exo_/C_cyt_ orientation, whereas MRAPs with the AP sequence at either end were found in dual N_exo_/C_cyt_ and N_cyt_/C_exo_ orientations. MRAP but not MART1 increased surface expression of co-expressed MC2 receptor (Figure 3B). MART1 was then tested in an experiment identical to that shown in Figure 2 for MRAP. AP–MART1 was expressed strongly but labeled only by ER-localized BirA_ER_ and labeling was rather weak (Figure 3C). By contrast, MART1–AP was expressed weakly but labeled strongly by cytoplasmic BirA and slightly by BirA_ER_. Labeling by BirA_ER_ probably occurred because of incomplete import of BirA_ER_ into the ER, which left some biotin ligase in the cytoplasm.

### Does MRAP Change Orientation after Biosynthesis?

**Figure 4:**
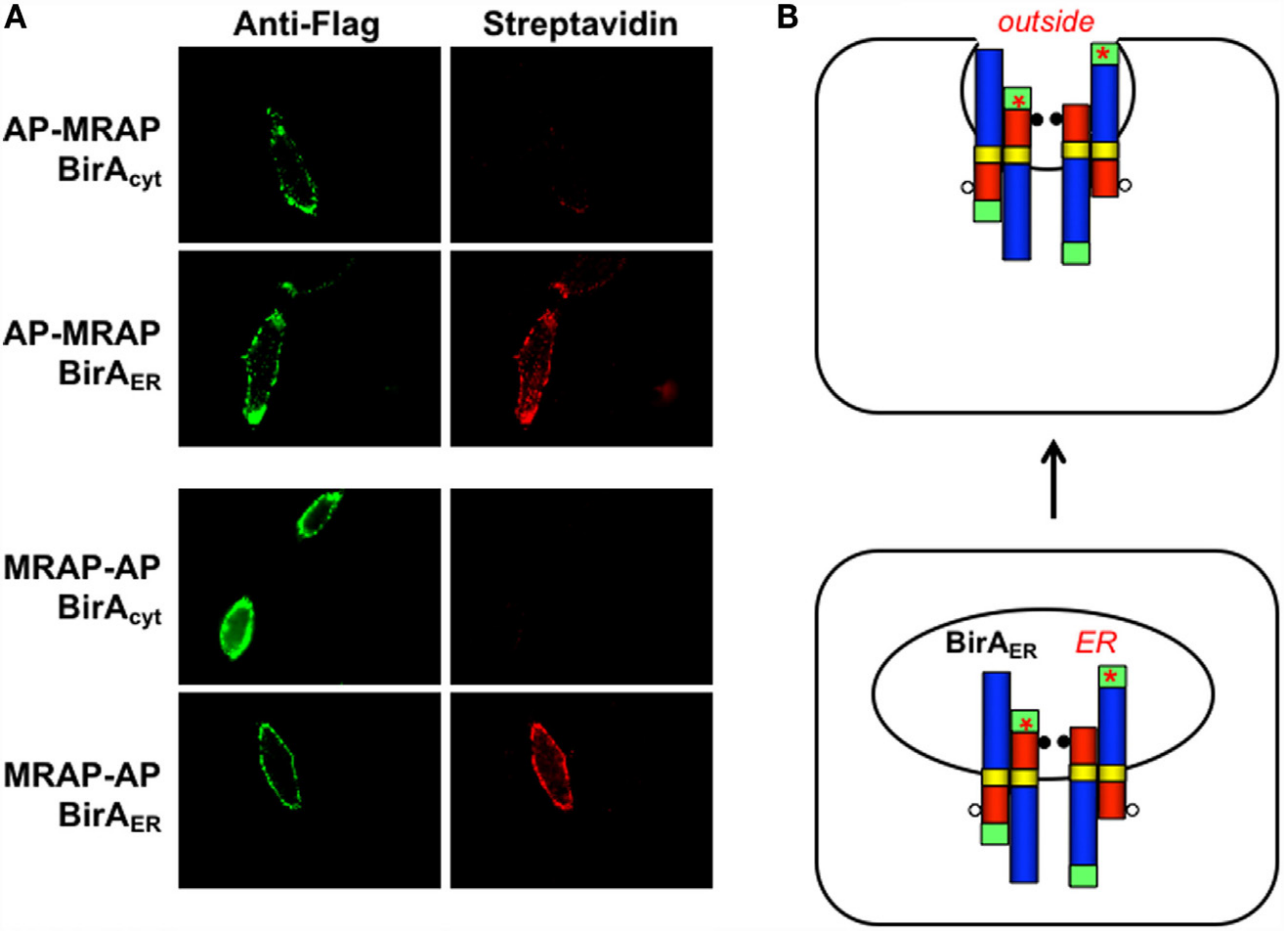
**Only ER-targeted BirA labels MRAP on the outer surface of the plasma membrane**. **(A)** CHO cells stably expressing BirA_cyt_ or BirA_ER_ were grown on coverslips and transiently transfected with plasmids encoding AP–MRAP or MRAP–AP, each containing a Flag epitope in the C-terminal domain. Live cells were incubated with monoclonal anti-Flag epitope followed by Alexa488-labeled anti-mouse IgG (green) and Alexa555-labeled streptavidin (red), washed and imaged. Barely visible untransfected cells not expressing MRAPs demonstrate low background staining. **(B)** Schematic representation of N_exo_/C_cyt_ and N_cyt_/C_exo_ MRAPs before and after trafficking to the plasma membrane assuming that orientation does not change.

To isolate MRAP that had trafficked to the plasma membrane, we localized cell surface biotin-labeled MRAP by fluorescence microscopy. Cells stably expressing cytoplasmic or ER-targeted BirA were transiently transfected to express either AP–MRAP–Flag or MRAP–Flag–AP. After incubation with biotin, live cells were stained with monoclonal anti-Flag antibody or fluorescent streptavidin. Because MRAP forms an antiparallel structure, both AP–MRAP–Flag and MRAP–Flag–AP were detectable on the cell surface with anti-Flag antibody, as expected (Figure 4A). On the other hand, if AP–MRAP–Flag and MRAP–Flag–AP did not change orientations following biosynthesis, only ER-localized BirA_ER_ would be able to label acceptor peptide in the ER lumen and, after trafficking, the extracellular side of the plasma membrane (Figure 4B). This result was obtained, with cell surface MRAP clearly labeled in all configurations but cell surface biotin-labeled MRAP visible only in cells expressing BirA_ER_. The results of this experiment are not confounded by incomplete import of BirA_ER_ into the ER.

**Figure 5:**
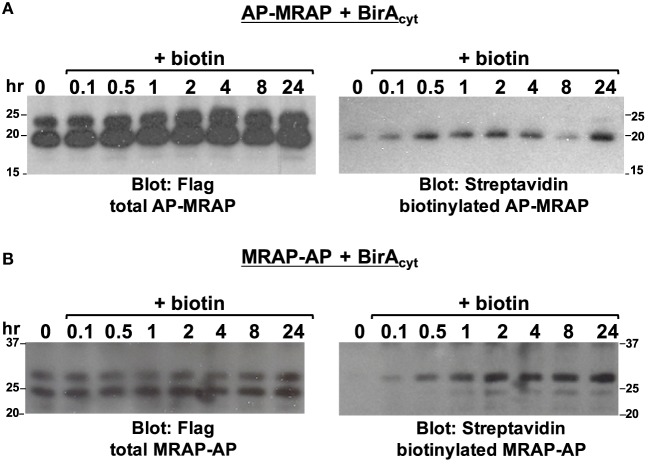
**Kinetics of biotin labeling**. CHO cells were transfected with BirA_cyt_ and either **(A)** AP–MRAP or **(B)** MRAP–AP, both containing Flag epitope. After overnight incubation in medium without added biotin, 2.5 μM biotin was added for the times shown. MRAPs were immunoprecipitated with anti-Flag antibody, resolved on SDS-PAGE and blotted with either anti-Flag antibody, to identify total MRAP, or HRP–streptavidin, to identify biotin-labeled protein.

A kinetic approach was also undertaken to determine whether MRAP changes orientation after biosynthesis (Figure 5). To avoid the ambiguity present in experiments using BirA_ER_, cells were transfected with cytoplasmic BirA and either AP–MRAP or MRAP–AP and incubated overnight in the absence of added biotin. During this period MRAP synthesis took place with very little biotin labeling (zero time point). Cells were then treated with added biotin for times up to 24 h. Immunoblotting for total AP–MRAP and MRAP–AP with anti-Flag antibodies is shown in the left panels. As seen previously (Figure 2A), cytoplasmic BirA labeled the faster-moving non-glycosylated AP–MRAP band. Increased biotin labeling could be detected as early as 6 min after biotin supplementation and labeling continued over time; after 24 h, there was only a trace amount of a higher molecular weight biotin-labeled AP–MRAP (Figure 5A). This result does not support the idea that MRAP flips orientations after synthesis. In the case of MRAP–AP, a minor fraction of non-glycosylated biotin-labeled MRAP was visible but the proportion did not increase with time, indicating that MRAP does not gradually flip but rather that a small amount of N_exo_/C_cyt_ MRAP does not become fully glycosylated (Figure 5B).

**Figure 6:**
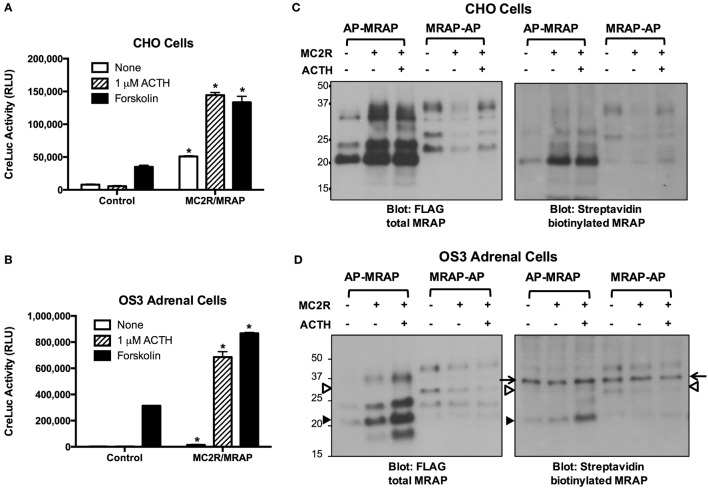
**MRAP orientation and function in CHO and adrenal cells**. **(A)** CHO and **(B)** OS3 adrenal cells were transfected with CRE-luciferase without (control) or with MRAP and MC2 receptor. The day after transfection cells were incubated for 5 h with vehicle, 1 μM ACTH or 20 μM forskolin when luciferase activity, expressed as relative light units (RLU), was measured. Note different scales for responses of OS3 and CHO cells. **(C)** CHO and **(D)** OS3 adrenal cells were transfected with BirA_cyt_ and AP–MRAP or MRAP–AP containing Flag epitope with or without MC2 receptor. Cultures were incubated overnight with or without 10 nM ACTH. Lysates were run on SDS-PAGE and blots were incubated with anti-Flag antibody to detect total MRAP or HRP–streptavidin to detect biotin-labeled MRAP. In **(D)**, the arrows point to a non-specific band, the solid arrowhead to non-glycosylated AP–MRAP, and the open arrowhead to glycosylated MRAP–AP. *P < 0.05 vs. corresponding value in cells not expressing MC2R/MRAP.

To characterize MRAP topology in its natural setting, we took advantage of the OS3 line of mouse adrenal cells that does not express MC2 receptors. ACTH responses were measured in parallel in OS3 and CHO cells transfected with the cAMP reporter CRE-luciferase with or without MC2 receptor and MRAP (Figures 6A,B). ACTH stimulated a 26-fold increase in the cAMP response in CHO cells expressing MRAP and MC2 receptor. Mock-transfected OS3 cells did not respond to ACTH, consistent with the reported absence of MC2 receptors (18). When the adrenal cells were transfected with MRAP and MC2 receptor, ACTH produced a striking 273-fold increase in cAMP reporter activity. OS3 cells must express MRAP, because MC2 receptors are functional when transfected alone. Interestingly, responses to forskolin were also exceptionally strong in OS3 cells. Forskolin acts directly on adenylyl cyclase and amplifies both basal and Gs-stimulated enzyme activity. Together, these findings suggest that adrenal OS3 cells have unusually efficient signal transduction at the G protein/adenylyl cyclase level.

We also explored MRAP orientation and the effects of MC2 receptor and ACTH (Figures 6C,D). OS3 cells and parallel cultures of CHO cells were transfected with BirA and AP-tagged MRAPs with or without MC2 receptors and incubated overnight with or without 10 nM ACTH. For reasons that are not clear, expression of AP–MRAP was higher when MC2 receptor was co-expressed and that of MRAP–AP was lower. ACTH had little effect. Biotin labeled the non-glycosylated AP–MRAP band, as predicted if topology does not change. In OS3 cells, there was a non-specific biotin-labeled protein at ~37 kDa that did not correspond to any MRAP band. MRAP–AP was weakly expressed in these cells and biotin-labeled species were partially obscured by the non-specific band.

### Is the MRAP Protein Stable?

**Figure 7:**
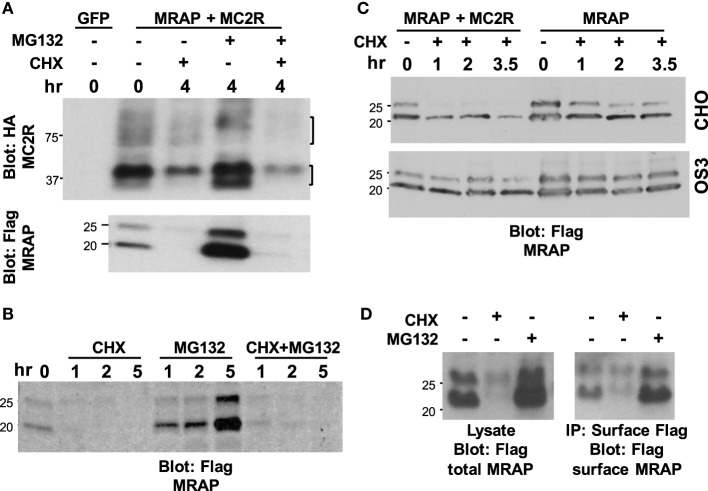
**Stability of MRAP and MC2 receptor**. **(A)** CHO cells stably expressing Flag-tagged MRAP were transfected with either GFP or HA-tagged MC2 receptor and then incubated for 4 h with no drug, 10 μg/ml cycloheximide (CHX), 50 μM MG132, or both. Cell lysates were run on SDS-PAGE and blotted for either *(upper)* MC2 receptor or *(lower)* MRAP. Brackets on the right show glycosylated MC2 receptor and core and non-glycosylated receptor. **(B,D)** CHO cells stably expressing Flag-tagged MRAP were incubated with 10 μg/ml cycloheximide (CHX), 50 μM MG132, or both for **(B)** times shown or **(D)** 1 h. In **(B)**, cells were lysed and immunoprecipitation with anti-Flag antibody performed whereas in **(D)** cells were incubated for 15 min with1:1000 anti-Flag antibody and washed extensively before lysis and collection of Flag-labeled proteins on Protein A/G beads to isolate cell surface MRAP. **(C)** CHO and OS3 adrenal cells were transiently transfected with MRAP or MC2 receptor and MRAP, then incubated with 100 μg/ml cycloheximide for the times shown, lysed and blotted for MRAP.

Having established that MRAP topology is stable, we examined the overall stability of the accessory protein. The stability of MRAP was estimated in CHO cells stably expressing Flag-tagged MRAP together with GFP or MC2 receptor. Cultures were treated for 4 h with the protein synthesis inhibitor cycloheximide (CHX), the proteasome inhibitor MG132 or both (Figure 7A). MRAP was quite labile, with little protein remaining 4 h after protein synthesis blockade. Surprisingly, MG132 by itself caused an enormous increase in the concentration of MRAP. On the other hand, MG132 did little to protect MRAP from degradation when CHX was present. A similar pattern was seen with the MC2 receptor, although the rate of degradation was slower (half-life between 3 and more than 8 h in different experiments) and effect of MG132 alone was much less marked. Mature MC2 receptor, which contains multiple sites for *N*-linked glycosylation (23, 24), ran as a broad band at high molecular weight, whereas immature core glycosylated and non-glycosylated receptor ran in more distinct bands close to the predicted molecular weight of 3xHA-hMC2 receptor, 37.3 kDa. Figure 7B shows a similar experiment demonstrating the rapidity of CHX and MG132 effects. The half-life of MRAP was estimated in similar experiments in which CHO cells were incubated with CHX from 0 to 5 h and MRAP levels were quantified densitometrically in immunoblots assuming first order kinetics. In most experiments, glycosylated MRAP was lost less rapidly than the non-glycosylated form, but this was not entirely consistent. The half-life of total MRAP averaged 1.7 ± 0.5 h (*n* = 7). The half-life of MRAP was short when it was transiently expressed with or without MC2 receptors in CHO cells but longer in adrenal OS3 cells, which grow less rapidly than CHO cells (Figure 7C). To study turnover of MRAP on the plasma membrane, CHO cells were incubated for 1 h with protein synthesis or proteasome inhibitors and then either lysed or incubated with antibody to label cell surface MRAP selectively (Figure 7D). The effects of CHX and MG132 were not as extreme but clearly present when plasma membrane MRAP was isolated.

**Figure 8:**
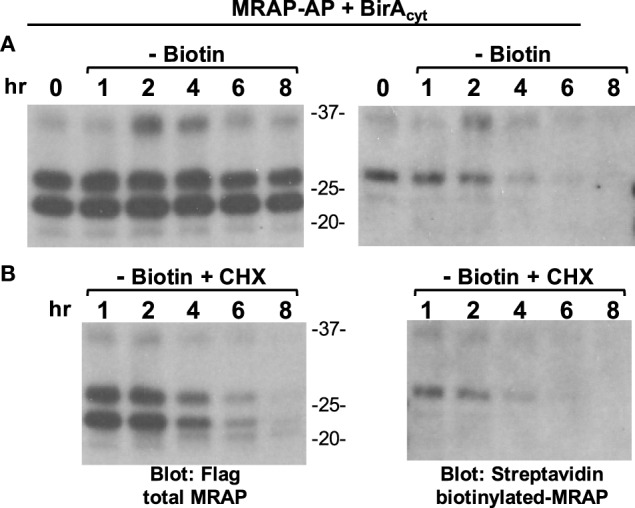
**Half-life of biotinylated MRAP is short with or without cycloheximide**. **(A,B)** CHO cells stably expressing BirA_cyt_ were transfected with MRAP–AP containing a Flag sequence, and incubated overnight in medium supplemented with biotin. Cells were then washed twice and incubated for 1–8 h in medium lacking biotin, **(A)** without or **(B)** with 10 μg/ml cycloheximide (CHX), when lysates were prepared and proteins resolved on SDS-PAGE and blots probed for either Flag epitope or biotin. The glycosylated form of MRAP–AP is labeled by cytoplasmic BirA.

The use of a global protein synthesis inhibitor to study degradation of a particular protein is complicated by potential effects on proteins other than the one under study. To avoid this problem, we studied cells stably expressing cytoplasmic BirA and then measured the amount of biotinylated-MRAP–AP at intervals after the removal of extracellular biotin with or without addition of CHX (Figure 8). Since biotin cannot be removed from a protein until it has been thoroughly degraded, this approach is conceptually equivalent to a radiolabeling pulse-chase experiment. For a low abundance protein, the method has the advantage of labeling only the protein of interest that can be concentrated on streptavidin beads prior to analysis. A limitation is the delay caused by the time required for washout of intracellular biotin. Following biotin withdrawal, total MRAP pools were quite stable over 20 h while normal synthesis and degradation were ongoing. The half-life of biotin-labeled MRAP was only 2.8 h, however, indicating that MRAP has a short half-life. In the presence of CHX, the corresponding values were 2.8 h for the total MRAP pool and 2.2 h for biotin-labeled MRAP. Because the half-lives measured by metabolic labeling and protein synthesis inhibition were strikingly similar, rapid turnover of MRAP was not an indirect effect of CHX.

**Figure 9:**
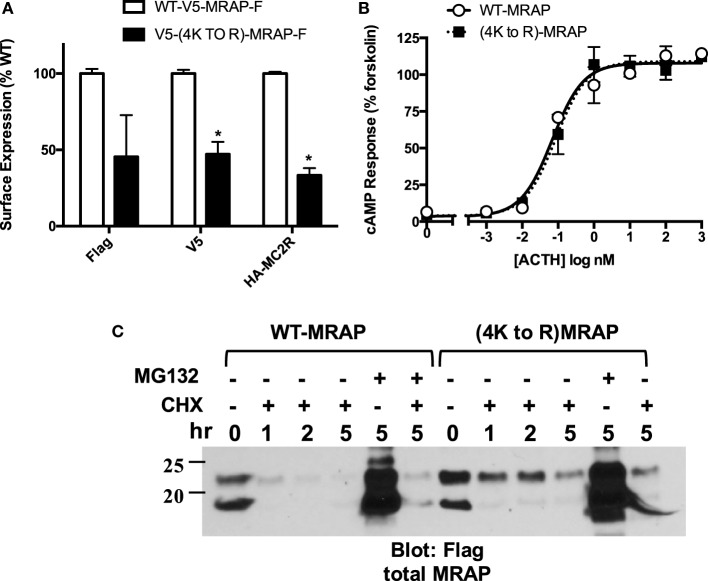
**Importance of MRAP Lys residues for function, orientation, and stability**. CHO cells were transfected with no accessory protein, wild-type MRAP or (4K to R)MRAP with the four native Lys residues mutated to Arg. **(A)** Surface expression of the N-terminal V5 and C-terminal Flag epitopes of MRAP and (4K to R)MRAP, and surface expression of co-transfected HA-tagged MC2 receptor. Results are normalized to values with wild-type MRAP. **(B)** cAMP responses to ACTH in cells co-transfected with MC2 receptor and CRE-luciferase. **(C)** Cells expressing MRAP or (4K to R)MRAP were incubated for the times shown with 10 μg/ ml cycloheximide (CHX), 50 μM MG132 or both. Lysates were run on gels and blotted for Flag epitope. *P < 0.05 vs. corresponding value in cells expressing wild-type MRAP.

In light of the effect of MG132, we investigated the possibility that MRAP itself undergoes ubiquitin-mediated degradation. In the canonical pathway ubiquitin is added to Lys residues and mouse MRAP has only four lysines, all located on the *N*-terminal side of the transmembrane helix in the juxtamembrane region. To test the importance of Lys to overall MRAP stability, we mutated these four residues to Arg (4K to R), retaining positive charge but removing lysine ɛ-amino groups that can be ubiquitylated. Arg-substituted MRAP was tagged with a V5 epitope on the *N*-terminus and a Flag epitope on the C-terminus, enabling analysis of its topology. Although (4K to R)MRAP was not expressed as highly as wild-type MRAP, it still assumed dual topology with both V5 and Flag epitopes on the plasma membrane (Figure 9A). Despite a lower level of MC2 receptor on the plasma membrane, cells expressing (4K to R)MRAP responded to ACTH with a cAMP increase equivalent to that supported by wild-type MRAP (Figure 9B). MG132 by itself caused an enormous increase in (4K to R)MRAP concentration just as it did with wild-type. Replacing all of the MRAP Lys residues with Arg delayed MRAP degradation by at most threefold (Figure 9C).

**Figure 10:**
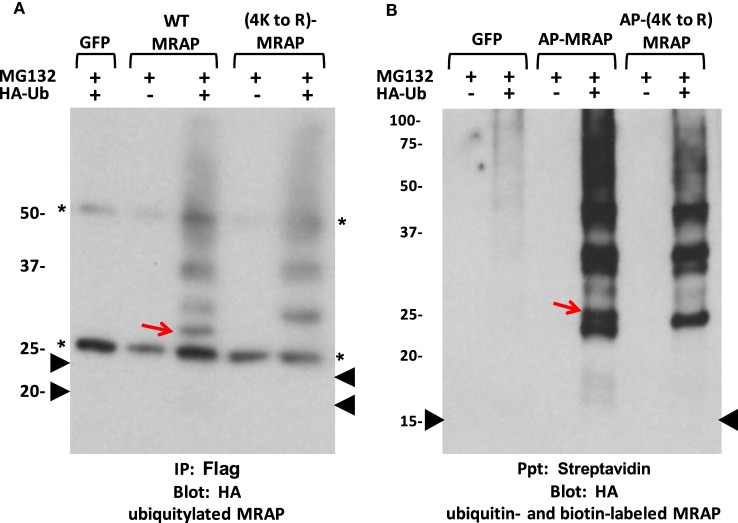
**MRAP is ubiquitylated**. **(A)** CHO cells were transfected with plasmids encoding GFP, Flag-tagged MRAP or (4K to R)MRAP, with or without HA-labeled ubiquitin (HA-Ub). After overnight incubation, cells were treated for 3 h with 50 μM MG132. MRAP proteins were immunoprecipitated with anti-Flag antibody and resolved on SDS-PAGE; blots were probed for HA. Asterisks mark IgG bands and black arrowheads show the position of the two MRAP bands from a parallel gel blotted for Flag. **(B)** CHO cells stably expressing cytoplasmic BirA were transfected with plasmids encoding GFP or AP–MRAP or AP-(4K to R)MRAP, both lacking any epitope tag, with or without HA-ubiquitin (HA-Ub). After overnight incubation in biotin-supplemented media, 50 μM MG132 was added for 3 h. Biotin-labeled proteins were collected on streptavidin beads and resolved on gels; blots were probed for HA. The arrowhead shows the position of biotin-labeled non-glycosylated AP–MRAP found on a parallel gel probed with streptavidin. The red arrows point to bands present in WT but not Lys-substituted MRAP. AP–MRAPs without epitope tags run at lower MWs than their longer tagged counterparts.

We tested whether ubiquitin is directly added to MRAP lysines by co-expressing HA-ubiquitin with either wild-type or (4K to R)MRAP in CHO cells and incubating for 3 h with MG132 to allow accumulation of any ubiquitylated species. Co-immunoprecipitation and immunoblotting revealed that Flag-tagged MRAP undergoes ubiquitin modification (Figure 10A). Multiple ubiquitylated MRAP bands were observed and only one of them was lost in the Arg-substituted mutant, suggesting that ubiquitin is added to Lys and also to one of the amino acids less frequently ubiquitylated: the aminoterminal amino group, the hydroxyl group of Ser or Thr, or the sulfhydryl group of Cys (25). On average, ubiquitylated MRAP species totaled ~5% of total MRAP after incubation for 3 h with a proteasome inhibitor.

To rule out the possibility that MRAP ubiquitylation was occurring on one of the Lys residues in the Flag sequence, we tested AP–MRAP and AP-(4K to R)MRAP that contained the biotin acceptor peptide sequence but no epitope tag in cells expressing cytoplasmic BirA (Figure 10B). Once labeled with biotin, the AP tag has no lysine amino group available for ubiquitin addition. Once again, multiple ubiquitylated MRAP bands were observed and only one was lost in the Arg-substituted mutant, suggesting that ubiquitin was added to MRAP at an atypical site.

**Figure 11:**
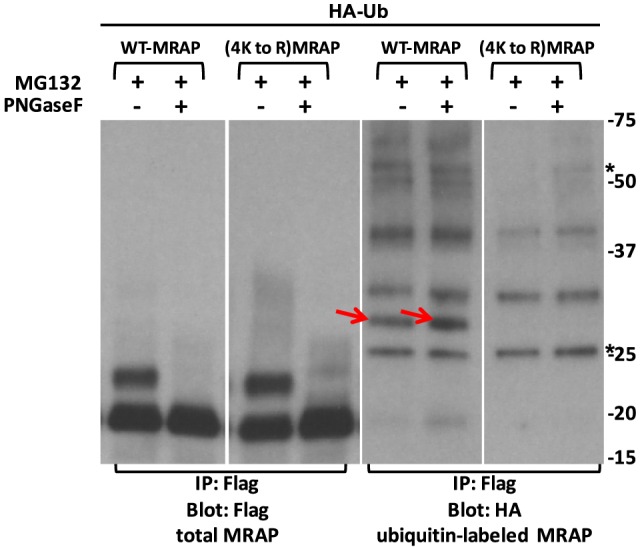
**Major ubiquitylated MRAP species are not glycosylated**. CHO cells were transfected with HA-ubiquitin (HA-Ub) and wild-type or (4K to R)MRAPs, which contained Flag epitopes. After overnight incubation cultures were treated for 3 h with 50 μM MG132 when lysates were prepared. Flag-tagged proteins were immunoprecipitated and treated with or without PNGaseF, which collapsed the slower-running glycosylated MRAP and (4K to R)MRAP bands. Asterisks show IgG bands. With longer exposure, higher MW bands were visible in the anti-Flag blot but obscured by the major MRAP bands. Red arrows point to ubiquitin-labeled bands present in WT but not Lys-substituted MRAP.

The glycosylation status of various MRAP bands was analyzed in lysates of cells expressing HA-Ub and MRAP before and after enzymatic deglycosylation with PNGaseF. None of the ubiquitylated MRAP bands was lost following PNGaseF treatment, whereas the slower-migrating MRAP bands collapsed to the mobility of the faster-migrating non-glycosylated species (Figure 11).

## Discussion

The unusual antiparallel dimer structure of MRAP been described only for MRAP and its paralog MRAP2. These two proteins are evolutionarily ancient (26–28) and have retained features that result in dual topology, including highly conserved aminoterminal and transmembrane domains and basic residues in the juxtamembrane region (29). The experiments described here do not explain what advantages are conferred by the conserved MRAP structure but they do establish that MRAP topology is stable from the time of MRAP biosynthesis to the time of MRAP degradation.

It has been particularly difficult to study MRAP topology because each side of the protein faces each direction in the antiparallel homodimer. Many of the methods used to assign membrane orientation require the addition of bulky fluorescent proteins or enzymes that are themselves larger than MRAP. Biotin labeling avoids this issue but has other drawbacks. Analysis of biotin-labeling patterns depended on the ability to distinguish between N_exo_ and N_cyt_ MRAPs based on glycosylation. Protein glycosylation never reaches 100%, and in our experience the extent of MRAP glycosylation varies with MRAP species, epitope tags, and expression levels. In addition, some BirA activity appeared to remain in the cytoplasm when ER-targeted BirA was overexpressed. Finally, backgrounds on HRP–streptavidin blots tended to be high when low abundance proteins were being detected. Despite these limitations, some situations were unambiguous. Once it has left the ER and Golgi apparatus, MRAP cannot undergo further glycosylation. Once it has been biotinylated by cytoplasmic BirA, MRAP cannot be orientated with biotin facing the outside of the cell unless it flips. The important point is that changes in the orientation of MRAP were not observed in any of configurations examined.

When protein synthesis was blocked, MRAP protein levels declined with a half-life that varied in different experiments but averaged under 2 h. Use of biotin labeling to study MRAP stability was again instructive, confirming that rapid MRAP turnover was not an artifact of CHX treatment. Although it is not known how quickly intracellular biotin declines when biotin is removed from the medium, this must happen quickly because biotin-labeled MRAP disappeared at roughly the same rate as total MRAP during protein synthesis blockade. A biotin-labeling approach has the potential to measure the half-life of any protein that can be modified by the addition of a biotin ligase acceptor peptide. It avoids the cost and risk of radioisotopes, is applicable for proteins with amino acid compositions that render them unsuitable for ^35^S-Met/Cys labeling, and can be used for proteins expressed at low abundance because of the ease of enriching on streptavidin beads.

Despite the presence of an array of chaperones that assist with protein folding in the ER, it is believed that a sizable fraction of newly synthesized protein fails to fold or assemble correctly. The accumulation of misfolded proteins in the ER can have disastrous outcomes, preventing synthesis of other membrane or secretory proteins. Cells contain elaborate and essential systems to remove terminally misfolded proteins from the ER, a process termed ER-associated degradation or ERAD (30, 31). ERAD involves recognition of misfolded protein, retrotranslocation from the ER, polyubiquitylation, and proteasomal degradation. Proteins are deglycosylated prior to degradation.

The involvement of proteasomal pathways in MRAP degradation was investigated here. One puzzling finding was that MG132 caused a very striking increase in the amount of MRAP when protein synthesis was ongoing but provided almost no stabilization when protein synthesis was blocked. Assuming that MG132 effectively inhibited proteasome activity, the results indicate that an alternative pathway for MRAP degradation exists and is responsible for the majority of MRAP degradation. The ability of MG132 to increase MRAP may be explained if the proteasome inhibitor increases the efficiency of MRAP biosynthesis, perhaps by inducing molecular chaperones (32).

Although the results with MG132 suggest that proteasomal degradation is not the major pathway for MRAP turnover, ubiquitylated MRAP was detected following a 3-h incubation with MG132. One ubiquitylated MRAP band had a molecular weight 8–10 kDa higher than the comparable non-glycosylated MRAP band and was lost in Arg-substituted MRAP, indicating that it represents MRAP with a single ubiquitin added to a lysine. At least two heavier ubiquitylated MRAP bands were present in MRAP lacking any lysines and must, therefore, represent ubiquitylation at some other amino acid but based on their migration on gels, these did not form a classical ubiquitin ladder. Cooray et al. demonstrated that MGRN1, an E3 ubiquitin ligase implicated in MC1 receptor function, is present in adrenal fasciculata and capable of adding ubiquitin to the MC2 receptor. They did not detect ubiquitylation of human MRAP in HEK293 cells in the absence of a proteasome inhibitor (24). Taken together, published work and the experiments described here indicate that polyubiquitylation and proteasomal degradation do not account for most MRAP degradation.

The stability of MRAP may be of interest in tissues outside of the adrenal gland. MC2R mRNA is highly expressed in adrenal and testicular tissue, whereas MRAPα mRNA has a somewhat broader distribution with high levels in the adrenal gland and testes but substantial amounts in fat and brain, among other tissues (3, 7). To date, there is no information about the relative expression of the protein products of these genes. hMRAPα coprecipitates with all five melanocortin receptors (5) and has modest effects on signaling by MC1 and MC3–5 receptors (7), making it plausible that MRAP has a role outside of the adrenal cortex. Patients who have glucocorticoid deficiency due to mutations in MC2 receptors and those who have mutations in MRAP display rather similar phenotypes (33), but comparison of MC2 receptor and MRAP knockout mice may provide insight into non-adrenal actions of MRAP in the future. A paralog of MRAP termed MRAP2 is widely distributed and influences signaling by MC4 receptors (34, 35) and at least one receptor outside the melanocortin receptor family (36) to regulate energy metabolism.

Not only is it uncertain how many G protein-coupled receptors interact with MRAPs, it is also uncertain whether MRAP actions are limited to receptors. The work detailed above supports previous findings of an antiparallel homodimer structure for MRAP and indicates that partners in the MRAP dimer remain in a fixed orientation during trafficking and expression on the plasma membrane. The finding that MRAP turns over rapidly in CHO cells needs to be interpreted cautiously because of the heterologous cell system, protein overexpression, and likely imbalance of accessory protein and receptor. An important question that remains to be addressed is whether MC2 receptor and MRAP protein levels change on a rapid time scale *in vivo*. Fortunately, ever more powerful mass spectrometry techniques are being developed and information about the stability membrane signaling proteins in a native environment should be forthcoming in the near future.

## Author Contributions

ZM, LJ, SM, and PH designed and conducted experiments, and PH wrote the manuscript.

## Conflict of Interest Statement

The authors declare that the research was conducted in the absence of any commercial or financial relationships that could be construed as a potential conflict of interest.

## Abbreviations

AP, acceptor peptide; BirAcyt, cytoplasmic biotin ligase; BirAER, ER-targeted biotin ligase-KDEL; Ccyt, cytoplasmic carboxyterminus; Cexo, extracellular carboxyterminus; CRE, cAMP response element; ER, endoplasmic reticulum; MC2R, melanocortin-2 receptor; MRAP, MC2 receptor accessory protein; Ncyt, intracellular aminoterminus; Nexo, extracellular aminoterminus.
